# A Trace Amount of Galactose, a Major Component of Milk Sugar, Allows Maturation of Glycoproteins during Sugar Starvation

**DOI:** 10.1016/j.isci.2018.11.035

**Published:** 2018-11-23

**Authors:** Norio Sasaoka, Hiromi Imamura, Akira Kakizuka

**Affiliations:** 1 Laboratory of Functional Biology, Graduate School of Biostudies, Kyoto University, Kyoto 606-8501, Japan

**Keywords:** Physiology, Cell Biology

## Abstract

Milk sugar is composed of glucose and galactose. Galactose is less suitable as an energy source than glucose. Thus, it has been a puzzle as to why mammals utilize galactose as a major component of milk sugar. Here we show that in hypoglycemic conditions, the presence of a trace amount of galactose, but not glucose, is able to maintain the production of mature glycoproteins and to abolish cell-death-inducing endoplasmic reticulum stress. In severely sugar-limited conditions, both glucose and galactose enter into the glycolytic pathway, but galactose is not able to raise the phosphofructokinase 1 activity, leading to the accumulation of fructose-6-phosphate, which in turn is utilized for the maturation of glycoproteins (e.g., growth factor receptors) and allows the activation of their intracellular signaling and prevents cell death from hypoglycemic conditions. Thus trace amounts of galactose may play unexpectedly important roles in the growth of infants and their protection during starvation.

## Highlights

• Trace amounts of galactose, but not glucose, allow maturation of glycoproteins
• Trace amounts of galactose, but not glucose, prevent ER stress and cell death
• Trace amounts of galactose raise F6P levels for the maturation of glycoproteins

## Graphical Abstract

**Figure:**
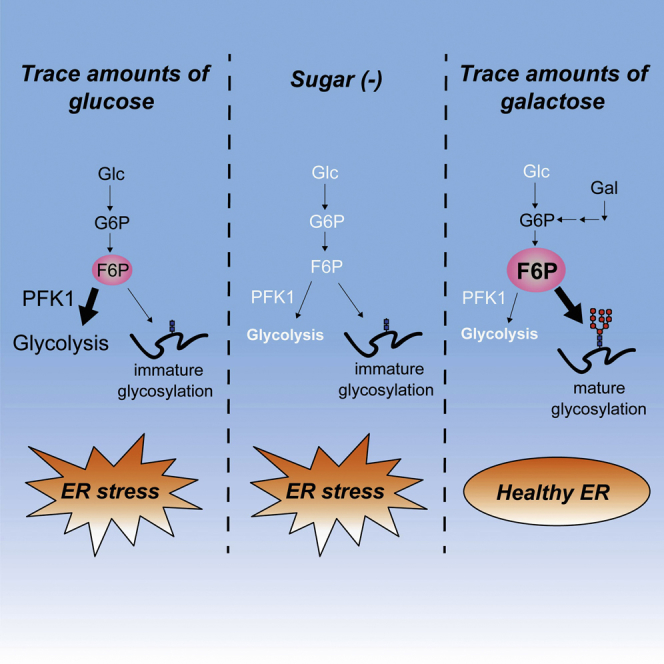

## Introduction

Maintenance of glucose levels is of great physiologic importance, and imbalances lead to an array of detrimental effects. Reduced glucose levels cause symptoms ranging from mild discomfort to severe confusion, seizures, and ultimately, brain damage (Agrawal et al., 2014). At the cellular level, glucose is mostly utilized as an energy source and is also required for protein glycosylation, which serves a variety of structural and functional roles in membrane-bound and secreted proteins. Glycosylation is a major form of protein modification in eukaryotes. It has been estimated that approximately half of all human proteins are glycosylated and most contain *N*-glycan structures (Apweiler et al., 1999). High-mannose-type *N*-glycans are reported to constitute approximately 40% of all *N*-glycans in mouse brains (Ishii et al., 2007). Furthermore, it is well known that *N*-glycosylation improves protein stability and regulates the function of cytokines and chemokines (Opdenakker et al., 1995). Hence, protein glycosylation plays very important roles for protein homeostasis and function.

Sugar insufficiency is one of the leading causes of endoplasmic reticulum (ER) stress. Cells cope with ER stress via three types of ER stress sensors and respond by deploying the unfolded protein response (UPR). The sensors are PKR-like ER kinase (PERK), inositol-requiring enzyme 1α (IRE1α), and activating transcription factor 6 (ATF6). Following the activation of these sensors, different signaling nodes, such as c-Jun N-terminal kinase 1, and downstream transcription factors, such as X-box-binding protein 1 (XBP1), activating transcription factor 4 (ATF4), and C/EBP homologous protein (CHOP), are activated (Mori, 2009). For example, IRE1α is activated by self-phosphorylation and manifests endoribonuclease activity, which cleaves a cryptic 26-bp exon from the downstream target *XBP1* mRNA. The resulting unconventionally spliced mRNA encodes an alternate XBP1 protein, spliced XBP1 (XBP1s), which is a highly active transcription factor that promotes the expression of ER chaperones and molecules involved in ER-associated degradation (Yoshida et al., 2001). Notably, XBP1s-dependent activation of the hexosamine biosynthetic pathway (HBP), which converts glucose to uridine 5′-diphosphate *N*-acetylglucosamine (UDP-GlcNAc) for *N*- and *O*-glycosylation of proteins, has been reported to protect cells from various biological insults, such as ER stress and cardiac ischemia/reperfusion injury (Wang et al., 2014). Hence, XBP1s is particularly important for cell survival.

Most studies of mammalian sugar metabolism concentrate on glucose, owing to its central roles in not only energy generation but also protein glycosylation. Other hexoses, e.g., mannose and galactose, receive relatively little attention in metabolic and physiologic studies. Mannose is a C2 epimer of glucose and is naturally found in microbes, plants, and animals. Free mannose is found in mammalian plasma at 50–100 μM (Alton et al., 1998). Galactose is a C4 epimer of glucose. Galactose, which is of particular relevance to mammals, is mostly produced by hydrolysis of the milk sugar lactose, a disaccharide with a glycosidic linkage between glucose and galactose. Free galactose concentrations in plasma are usually <10 mg/dL (0.56 mM) (Kaempf et al., 1988). The detectable plasma galactose levels in normal adult humans suggest that the dietary acquisition of modest amounts of galactose from fruits and vegetables, e.g., tomatoes, bell peppers, brussels sprouts, apples, bananas, persimmons, dates, etc., (Gross and Acosta, 1991) is beneficial. Galactose is metabolized by the enzymes of the Leloir pathway in the liver (Frey, 1996), which requires several enzymes to convert galactose to glucose-6-phosphate (G6P). In mammals, mutations in some of these enzymes can result in the genetic disease galactosemia (Leslie, 2003, Holden et al., 2004, Timson, 2006). Despite such disadvantageous features of galactose, mammals must use galactose for something important, because lactose, the milk sugar, consists of galactose and glucose as a heterodimer. Based on the evidence that lactose is an essential nutrient for mammalian babies, one of the biggest and long-lasting questions in biology has been why galactose has been evolutionally selected as a major component of lactose.

## Results

### Galactose Promotes *N*-Glycosylation Preferentially over Glucose or Mannose

**Figure 1:**
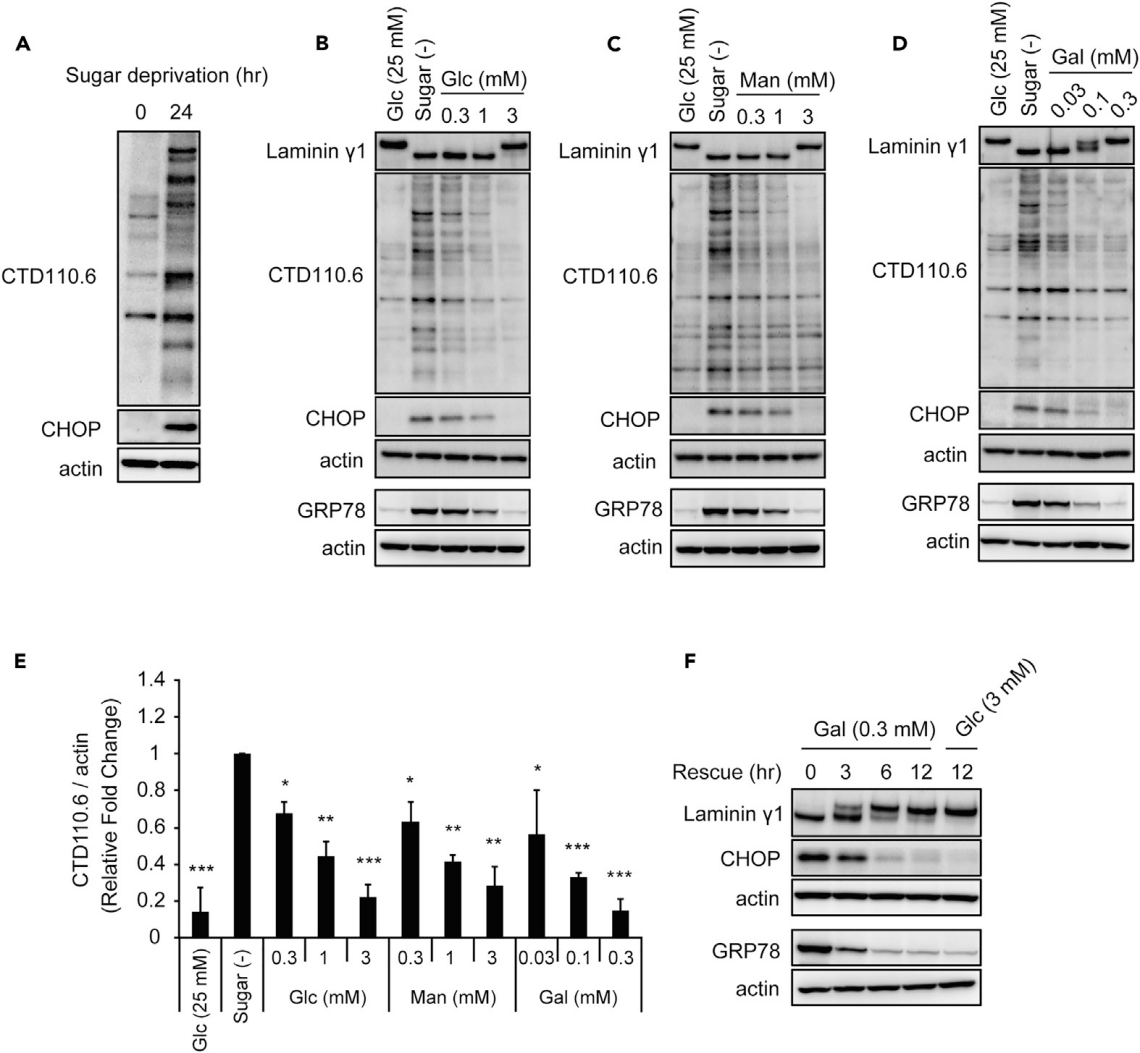
Galactose Promotes *N*-Glycosylation Preferentially over Glucose or Mannose (A) HEK293 cells were deprived of glucose for 24 hr. Total cell lysates were analyzed by western blot with CTD110.6 to assess *N*-glycosylation levels and an antibody against CHOP as an ER stress marker. (B–D) The promoting effect of glucose (B), mannose (C), or galactose (D) for *N*-glycosylation. Each immunoblot is represented by laminin γ1, CTD110.6, CHOP, and GRP78. (E) Quantitative analysis of reactivity with CTD110.6 antibody in (B–D), normalized to actin intensity. The value for the sugar-free condition was set as 1. The data are the mean ± SEM (n = 3). *p < 0.05, **p < 0.01, ***p < 0.005 by Dunnett's test. (F) HEK293 cells were pre-treated with sugar-free medium for 24 hr followed by re-feeding 0.3 mM galactose for indicated times. Actin was monitored as a loading control. Cells were cultured under serum-free conditions. See also Figures S1–S3.

Sugar deprivation induces immature *N*-GlcNAc_2_-modified glycoproteins, which cross-react with the *O*-GlcNAc-specific antibody CTD110.6 (Isono, 2011). We also independently observed that HEK293 cells produced proteins recognized by CTD110.6 antibody under sugar deprivation and that ER stress was evident under this condition (Figure 1A). We successfully identified laminin γ1 and laminin β2 as CTD110.6-antibody-recognized proteins, by immunoprecipitation with CTD110.6 antibody, followed by liquid chromatography-tandem mass spectrometry analyses (Figures S1A and S1B). Both of these are *N*-linked high-mannose-type glycoproteins (Fujiwara et al., 1998). Indeed, CTD110.6 antibody recognized only lower-molecular-weight or immature forms of laminin γ1 and laminin β2, which appeared only upon sugar starvation. We also showed that an anti-laminin γ1 antibody revealed aggregate-like structures in cells under sugar starvation, most likely in the ER (Ikeda et al., 2014). Pulse-chase experiments using [^35^S]methionine/cysteine indicated that *N*-GlcNAc_2_-modified laminin β2 was a newly synthesized protein rather than a cleavage product of mature *N*-glycosylated laminin β2 (Figure S2). Addition of varying amounts of glucose in a dose- and time-dependent manner eliminated these immature glycoproteins and ER stress (Figures 1B and S1C). These results clearly indicated that sugar-deprivation-induced ER stress is correlated with the accumulation of immature glycoproteins in the ER.

To elucidate the role of other hexoses in protein glycosylation, we then focused on mannose and galactose and examined how these sugars function in producing mature *N*-glycosylated proteins, when compared with glucose. Mannose showed an essentially identical capacity as glucose with regard to the production of mature *N*-glycosylated proteins and the reduction of ER stress (Figure 1C). Surprisingly, however, galactose was 10 times more effective at producing mature *N*-glycosylated proteins and canceling ER stress (Figure 1D). In other words, 0.3 mM galactose was just as effective as 3 mM glucose or 3 mM mannose in the production of mature *N*-glycosylated proteins and abatement of ER stress (Figures 1B–1E). Furthermore, both 0.3 mM galactose (Figure 1F) and 3 mM glucose (Figure S1C) led to mature laminin γ1 production over a similar time course. We also observed that galactose had the same robust ability to produce mature *N*-glycosylated proteins and to reduce ER stress in other cell lines, including human hepatoblastoma-derived HepG2 cells and rat pheochromocytoma-derived PC12 cells (Figure S3).

### Sugar Deprivation Increases the Expression of Enzymes Involved in *N*-Glycosylation via XBP1s

**Figure 2:**
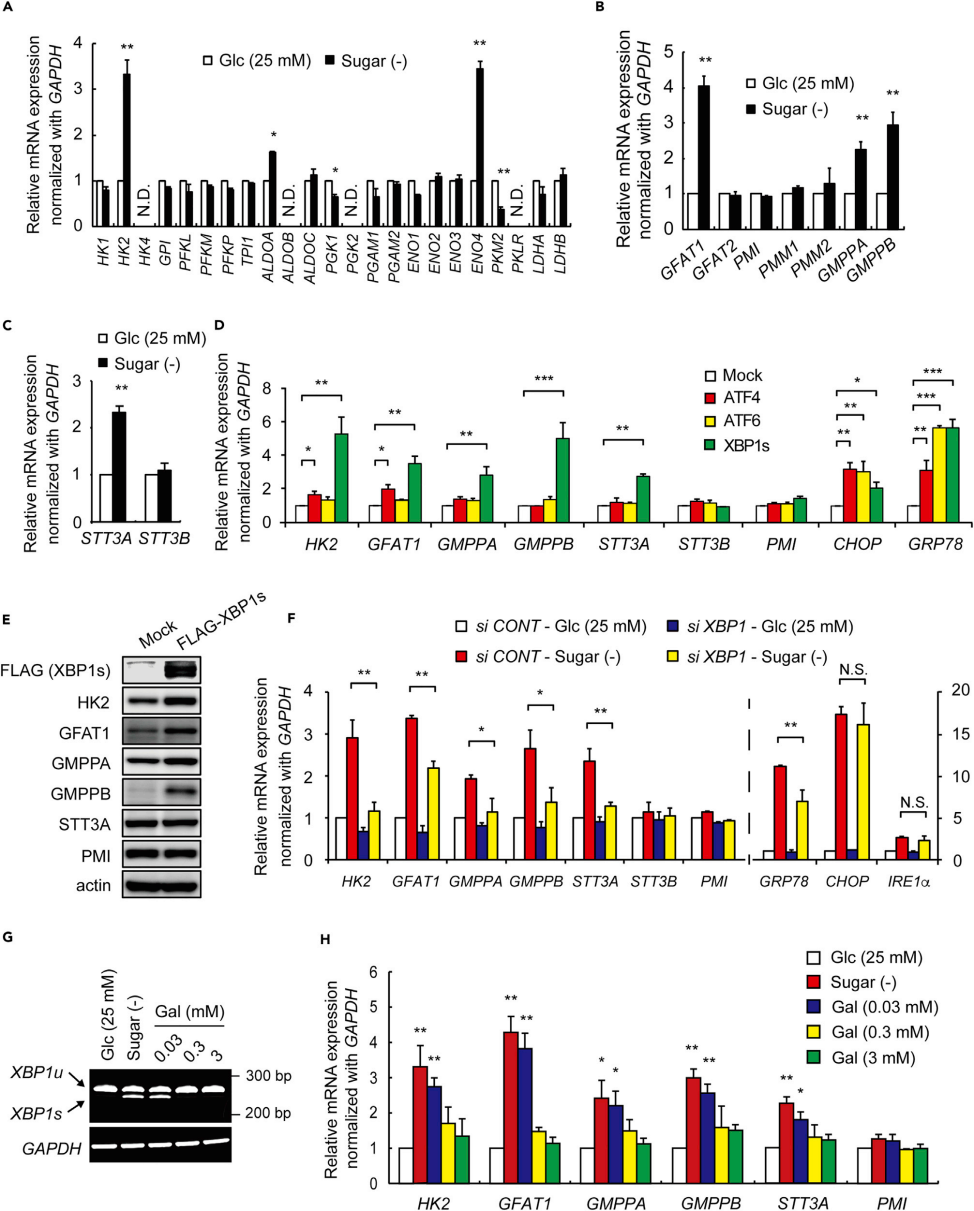
Galactose Cancels the Upregulation of *N*-Glycosylation-Related Genes Induced by Spliced XBP1 (A–C) Quantification of indicated gene transcripts by qRT-PCR. HEK293 cells were deprived of sugar for 24 hr, and gene transcripts in the glycolytic pathway (A), in the hexosamine biosynthetic pathway (B), and of the catalytic subunits of the *N*-oligosaccharyltrasferase complex (C) were analyzed. The values are the mean ± SEM (n = 3–6). N.D., not detected. *p < 0.05, **p < 0.01 by Student's t test. (D–F) Spliced XBP1 (XBP1s) increases glycosylation-related genes. (D) HEK293 cells were transfected with mock (white bars), ATF4 (red bars), ATF6 (yellow bars), or XBP1s (green bars) cDNA expression vectors, and cultured in 25 mM glucose condition for 24 hr. The expression levels of the indicated genes were measured by qRT-PCR, relative to the mock control, which was arbitrarily set as 1. Mannose phosphate isomerase (PMI) was included as a negative control. (E) HEK293 cells were transfected with FLAG-XBP1s and cultured in medium containing 25 mM glucose for 24 hr. Total cell lysates were analyzed by western blot. (F) Effect of *XBP1* knockdown on indicated *N*-glycosylation-related gene expression in HEK293 cells treated with or without sugar deprivation for 24 hr. *GRP78* as well as *CHOP* and *IRE1α* were used as positive and negative controls, respectively. The values are the mean ± SEM (n = 3–6). N.S., not significant. *p < 0.05, **p < 0.01, ***p < 0.005 by Dunnett's test. (G) XBP1 splicing assay. RNAs from HEK293 cells treated with the indicated conditions for 24 hr were examined by RT-PCR. (H) Galactose effects on the expression of *N*-glycosylation-related genes were analyzed by qRT-PCR. HEK293 cells were treated in conditions described in (G). *PMI* was used as a negative control. The values are the mean ± SEM (n = 3–6). *p < 0.05, **p < 0.01 by Dunnett's test. Cells were cultured under serum-free conditions. See also Figures S4–S6, Tables S1 and S2.

Activated nucleotide sugars, such as UDP-GlcNAc and guanosine diphosphate mannose (GDP-Man), which are required for *N*-glycosylation, are derived from fructose-6-phosphate (F6P) (Dennis et al., 2009). We therefore speculated that cellular mechanisms should exist to compensate for the glycosylation failure under sugar deprivation. Based on such speculation, we performed qRT-PCR analyses on mRNAs of glycolytic pathway enzymes and observed the upregulation of *HK2*, *ALDO*, and *ENO4* mRNAs in response to sugar deprivation (Figure 2A). We also observed upregulation of mRNAs for *GFAT1* and *GMPP* (*A* and *B*), which are responsible for the production of UDP-GlcNAc and GDP-Man, respectively (Figures 2B and 2C) (Dennis et al., 2009, Szumilo et al., 1993, Ning and Elbein, 2000). mRNA for *STT3A,* but not for *STT3B*, both of which encode catalytic subunits of the *N*-oligosaccharyltransferase (OST) complex (Kelleher et al., 2003, Ruiz-Canada et al., 2009), was also upregulated (Figure 2C). Protein levels of HK2, GFAT1, GMPPA, and GMPPB were also increased by sugar deprivation (Figure S4). Subsequently, we tested whether other ER stress inducers, e.g., thapsigargin and brefeldin A, produced similar effects and found that *HK2*, *GFAT1*, *GMPPA*, *GMPPB*, and *STT3A* mRNAs and the corresponding proteins increased (Figure S5).

Under ER stress, ATF4, ATF6, and XBP1 function as transcription factors to upregulate their target genes to cope with the ER-stress-inducing conditions; the induction of these specific target mRNAs constitutes part of the UPR (Walter and Ron, 2011). We examined whether overexpression of these transcription factors would alter the expression of the same genes that were upregulated during sugar deprivation. We found that overexpression of the ER-stress-related XBP1s led to increases in transcription of all the above-mentioned genes that were upregulated during sugar deprivation (Figure 2D); overexpression of ATF4, but not ATF6, just slightly increased the abundance of *HK2* and *GFAT1* mRNAs (Figure 2D). Overexpression of ATF4 or ATF6 increased the expression of *XBP1* mRNA in the glucose(+) condition (Figures S6A and S6B) (Yoshida et al., 2001, Tsuru et al., 2016). However, overexpression of ATF4, but not ATF6, induced IRE1α protein (Figure S6C), and the former, but not the latter, marginally induced the unconventional splicing that generated *XBP1s* mRNA, albeit very weakly (Figure S6D) (Tsuru et al., 2016). The *XBP1* splicing appeared to occur without inducing conspicuous IRE1α phosphorylation. This observation may be explained as follows: the increased IRE1α protein might weekly oligomerize by itself and induce self-phosphorylation, but the phosphorylation levels were very weak and below the detection levels of the antibody used. Overexpression of XBP1s, but not ATF4 or ATF6, clearly upregulated the protein levels of *N*-glycosylation-related genes, except for STT3A (Figure 2E). We also found that *XBP1* knockdown significantly diminished the upregulation of the *N*-glycosylation-related mRNAs in response to sugar deprivation (Figure 2F). Furthermore, *XBP1s* production, induced by sugar deprivation, was inhibited by the addition of galactose in a dose-dependent manner (Figure 2G). Consistent with these results, the addition of a trace amount of galactose dose dependently suppressed the upregulation of the *N*-glycosylation-related mRNAs that were induced by sugar deprivation (Figure 2H).

### Accumulation of F6P in Galactose(+) Condition

**Figure 3:**
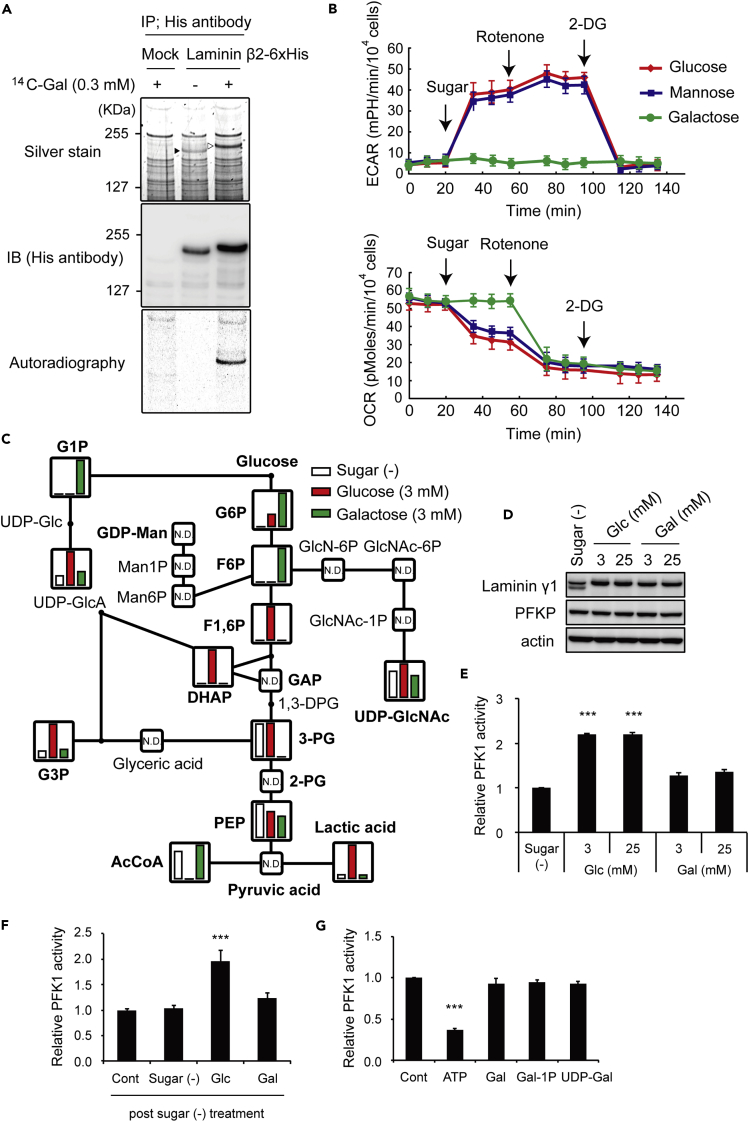
Alteration of Metabolism in Response to Galactose (A) Incorporation of [^14^C]galactose into laminin β2. HEK293 cells were transfected with mock or a C-terminal His-tagged laminin β2 expression construct, and were then cultured in medium with or without 0.3 mM [^14^C]galactose for 24 hr. Immunoprecipitates with His antibody were analyzed by silver staining, western blot, and autoradiography. (B) The response of ECAR (upper panel) and OCR (lower panel). HEK293 cells were stimulated with glucose (3 mM), mannose (3 mM), or galactose (3 mM), and subsequently subjected to the mitochondria inhibitor rotenone (3 μM) and the glycolysis inhibitor 2-DG (100 mM) at the indicated times. Both datasets are from the same experiment, and each data point represents the mean ± SEM, n = 4. (C) Metabolomic analysis of HEK293 cells treated with sugar-free (white), glucose (3 mM, red), and galactose (3 mM, green) conditions for 6 hr. The relative quantities of the annotated metabolites are represented as bar graphs. (D) Representative immunoblot of PFKP in response to the indicated treatments. (E) HEK293 cells were treated with the indicated conditions for 6 hr, and PFK1 activity was analyzed by measuring NADH consumption. (F) HEK293 cells were sugar-deprived (SD) for 6 hr, followed by restoration with the indicated sugars (3 mM each) for 1 hr. (G) HEK293 cells were cultured in medium containing glucose (3 mM) for 6 hr, and PFK1 activity was analyzed in a reaction mixture that included galactose (Gal) (3 mM), galactose-1-phosphate (Gal-1P) (3 mM), or UDP galactose (UDP-Gal) (3 mM). The data are the mean ± SEM. ***p < 0.005 by Dunnett's test. Cells were cultured under serum-free conditions. See also Figure S7.

We then questioned whether added galactose is metabolized via the Leloir pathway to activated nucleotide sugars and is used in the mature *N*-glycosylated proteins, such as laminin β2. We then observed that [^14^C]galactose was indeed incorporated in mature laminin β2 (Figure 3A), demonstrating that the added galactose is incorporated as a carbon source into sugar chains of laminin β2.

We next measured the extracellular acidification rate (ECAR) and oxygen consumption rate (OCR). Glucose or mannose immediately increased ECAR, leading to a compensatory decrease of OCR. In contrast, galactose did not alter ECAR or OCR at all (Figure 3B). Rotenone, a complex I inhibitor, decreased OCR with all indicated sugar conditions. Decreased OCR induced a compensatory increase of ECAR in glucose(+) or mannose(+) medium, but not in galactose(+) medium (Figure 3B).

Given that galactose did not affect ECAR despite entering the glycolytic pathway, we expected that in the galactose(+) medium the glycolytic pathway may arrest, and then the corresponding metabolites would accumulate somewhere within the glycolytic pathway. We therefore performed a metabolome analysis with capillary electrophoresis time-of-flight mass spectrometry (CE-TOFMS) to evaluate metabolite levels in HEK293 cells treated with sugar-free, 3 mM glucose, and 3 mM galactose for 6 hr (Figure 3C). In the sugar-free condition, levels of metabolites just upstream of dihydroxyacetone phosphate (DHAP) were essentially undetectable, e.g., glucose-1-phosphate (G1P), G6P, F6P, and fructose-1, 6-bisphosphate (Figure 3C). In the glucose(+) condition, the metabolites upstream of F6P appeared to be rapidly metabolized in the glycolytic pathway (Figure 3C). By contrast, in the galactose(+) condition, metabolite levels upstream of F6P dramatically increased, when compared with the sugar-free and the glucose(+) conditions. Consistent with the results of flux analyses (Figure 3B), in the galactose(+) condition, the amount of lactic acid, which reflects the ECAR, was comparable to that in the sugar-free condition, and was much lower than the amount of lactic acid in the glucose(+) condition (Figure 3C).

The level of UDP-GlcNAc even in the sugar-free condition was similar to those in the other two conditions (Figure 3C), and cells cultured in the sugar-free condition for a much longer time (e.g., 72 hours) retained the *N*-GlcNAc_2_ modifications (Figure S7A), indicating that UDP-GlcNAc is mostly supplied by its re-usage in the cells (Rome and Hill, 1986), but not by HBP. Consistently, cells cultured with azaserine or 6-diazo-5-oxo-1-norleucine, both of which are HBP inhibitors, were unaffected with regard to the maturation of *N*-glycosylated proteins or the abatement of ER stress in the presence of glucose or galactose (Figure S7B). These results further support the idea that accumulated F6P in the galactose(+) condition is preferentially utilized to produce GDP-Man for the maturation of high-mannose-type glycoproteins. It is notable that addition of bafilomycin A1 or E64d/pepstatin A, each of which is an inhibitor of autophagy, did not affect the maturation of *N*-glycosylated proteins by galactose (Figure S7C). These results appear to rule out the involvement of autophagy in the maturation of *N*-glycosylated proteins by galactose.

We next investigated whether galactose affects the expression level of phosphofructokinase 1 (PFK1), but the expression levels of PFK1 in the HEK293 cells were almost identical among all conditions (Figure 3D). We then compared PFK1 activity among the conditions. Interestingly, we found that PFK1 activity increased in parallel with the glucose concentrations of culture medium, and galactose was unable to activate PFK1 (Figures 3E and 3F). On the other hand, the PFK1 activity was not inhibited by the addition of galactose or galactose metabolites, e.g., galactose-1-phosphate and UDP galactose (Figure 3G). Taken together, these results indicate that galactose does not stimulate the PFK1 activity, leading to the accumulation of galactose-derived F6P.

### A Trace Amount of Galactose Reduces ER Stress and Cell Death in Sugar Deprivation

**Figure 4:**
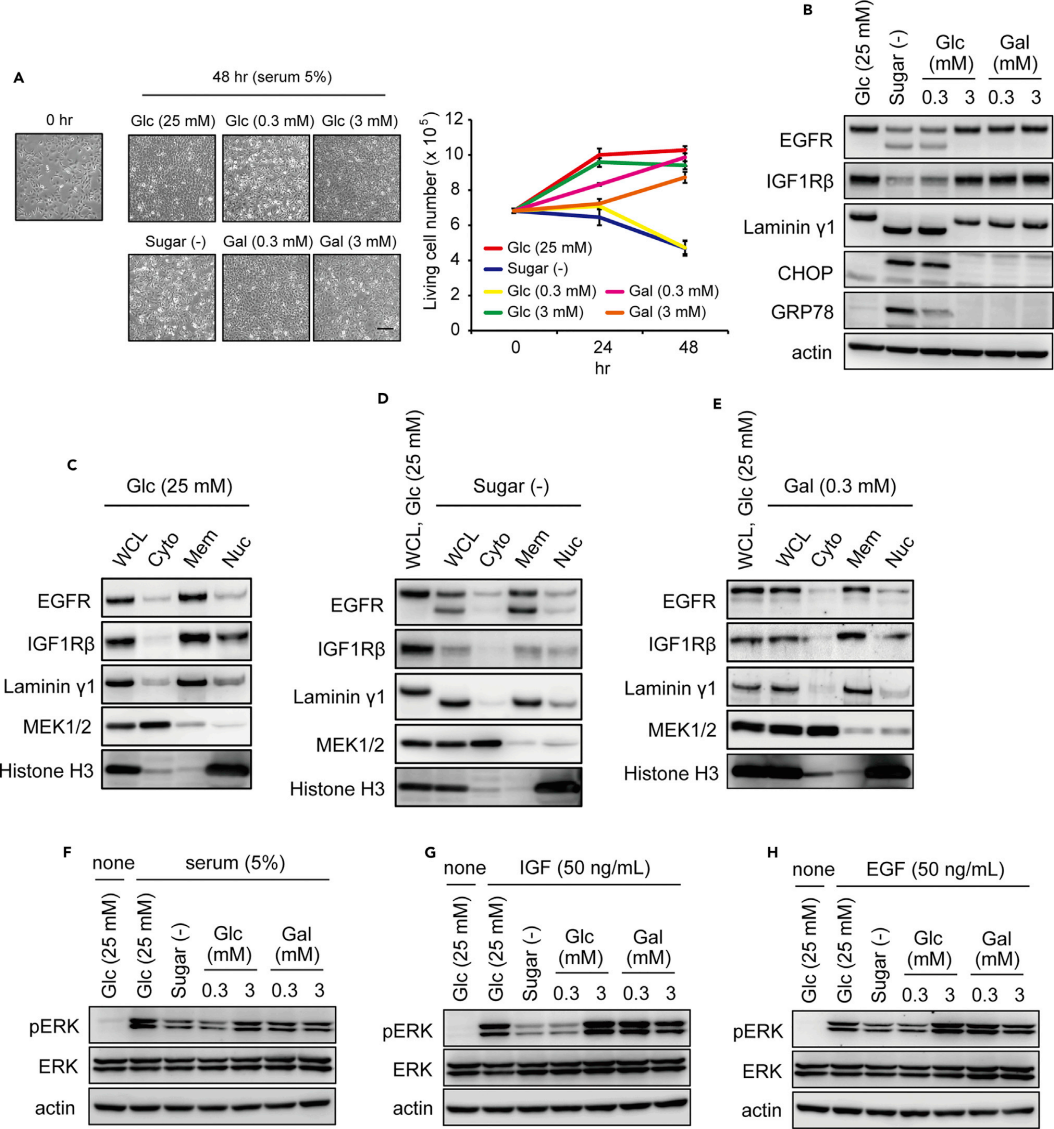
A Trace Amount of Galactose Promotes Cell Survival and Growth as well as Maturation of Growth Factor Receptors (A) Photographs of HEK293 cells, cultured with sugar-free, glucose, or galactose conditions for 48 hr, starting from cells at 70% confluency in each well of a 6-well plate at time 0 (left). Live cell numbers were counted by staining with trypan blue (right). The data are the mean ± SEM (n = 3). Scale bar, 50 μm. (B) Expression of IGF1Rβ and EGFR. HEK293 cells were treated with the indicated conditions for 24 hr, and whole-cell lysates were analyzed by western blot. (C–E) Cell fractionation to examine glycoproteins after growth in medium with (C) 25 mM glucose, (D) sugar-free, or (E) 0.3 mM galactose. Antibodies against EGFR, IGF1Rβ, and laminin γ1 were as in (B), and MEK1/2 and histone H3 were markers for the cytoplasmic and nuclear fractions, respectively. (F–H) ERK activation by extracellular stimulation. HEK293 cells were cultured with the indicated conditions without serum for 24 hr before stimulation with 5% serum for 5 min (F), 50 ng/mL IGF1 for 10 min (G), or 50 ng/mL EGF for 5 min (H). Western blot analysis detected phosphorylated and total ERK levels under different conditions of sugar supplementation, as indicated. Actin was used as a loading control. Cells in (B–H) were cultured under serum-free conditions.

Next, we investigated the effects of glucose or galactose on the prevention of cell death induced by sugar deprivation. Supplementation with 3 and 25 mM glucose increased cell proliferation in a dose-dependent manner (Figure 4A). However, 0.3 mM glucose showed no effect on cell proliferation or protection, when compared with the sugar-free condition. In contrast, 0.3 mM galactose dramatically induced cell proliferation. With 3 mM galactose, cell proliferation slightly decreased, compared with 3 mM glucose or 0.3 mM galactose. These observations may reflect a condition of patients with galactosemia, having a milieu of symptoms including cataracts, liver, and brain growth failure (Lai et al., 2009).

### A Trace Amount of Galactose Promotes Growth Signal Transduction via the Maturation of Protein Glycosylation

Based on the evidence that many growth factors and their receptors are glycoproteins (Takahashi et al., 2004, Kaszuba et al., 2015), we examined the protein status of growth factor receptors, such as epidermal growth factor (EGF) receptor (EGFR) and insulin growth factor (IGF) receptor (IGFR). In the sugar-starved conditions, maturation of EGFR was clearly inhibited, and protein levels of IGF1Rβ were greatly reduced (Figure 4B). Mature and immature EGFR and IGF1Rβ were mainly detected in the membrane fraction (Figures 4C–4E). However, in the starved condition, responses of cells to EGF, IGF, and serum were reduced, which was evidenced by the absence of ERK phosphorylation (Figures 4F–4H). Addition of a trace amount of galactose, namely, 0.3 mM galactose, was able to correct these abnormalities. By contrast, these abnormalities were not corrected by the addition of 0.3 mM glucose (Figures 4F–4H).

## Discussion

Animals must overcome various environmental challenges. Among them, starvation might be common but most dangerous, and thus animals should be equipped to cope with such potentially fatal conditions. Starvation, especially sugar deprivation, has been known to induce ER stress, which is evidenced by the induction of CHOP and GRP78 proteins. In this study, we observed the production of immature glycosylation forms of high-mannose-type glycoproteins, which were recognized by CTD110.6 antibody in sugar-starved cultured cells (Isono, 2011). Given that levels of GDP-Man decrease under sugar starvation, and that the molecule recognized by CTD110.6 antibody is *N*-GlcNAc_2_ (Isono, 2011), addition of mannose to dolichol-pyrophosphate-GlcNAc_2_ (Dol-PP-GlcNAc_2_) by β-1,4 mannosyltransferase-1 (ALG1) (Couto et al., 1984) would not occur. Thus ALG1 functions as the sensor to monitor the levels of GDP-Man in the cells; when the levels of GDP-Man become much lower than the KD value, any addition of mannose to Dol-PP-GlcNAc_2_ would cease. In this scenario, mammalian cells utilize such naked Dol-PP-GlcNAc_2_, instead of mature glycans, as the substrate of OST to produce immature glycoproteins. As the presence of such immature glycoproteins appeared to be intimately coupled with the evocation of ER stress, cells appear to engage a system to induce an ER stress response via the production of immature glycoproteins under starvation. Consistently, we were able to show that ER stress leads to the expression of HK2, GFAT1, and GMPP, essential enzymes involved in the synthesis of *N*-glycosylation components (e.g., UDP-GlcNAc, GDP-Man), and that XBP1s is responsible for their expression. It is noteworthy that UDP-GlcNAc and GDP-Man are also utilized in *O*-glycosylation (Spiro, 2002). Therefore, it is also expected that maturation of not only *N*-glycosylated but also *O*-glycosylated proteins is also hindered under sugar starvation and that maturation of both *N*- and *O*-glycosylated proteins is also restored by a trace amount of galactose.

Other ER stress inducers, e.g., thapsigargin and brefeldin A, which are not related to the production of immature glycoproteins, also induce *HK2*, *GFAT1*, *GMPPA*, *GMPPB*, and *STT3A* mRNAs and the corresponding proteins, indicating that the response to immature glycoproteins is a fundamental, perhaps archetypical ER stress response.

We demonstrated that galactose is much more potent to maintain mature *N-*glycosylation of proteins than glucose and mannose. Maintenance of mature *N-*glycosylation contributes to the relief of starvation-induced ER stress and cell death and also to appropriate growth-factor-mediated signal transduction. From our metabolome data, under the galactose(+) condition accumulation of galactose-derived F6P was observed, which would be utilized in the production of nucleotide sugars required for *N*-glycosylation. However, such F6P accumulation was not observed in the glucose(+) condition. It is notable that galactose itself is utilized in other types of glycosylation. These observations indicate that galactose from milk or milk sugar would preferentially contribute to produce mature glycoproteins rather than to energy production during starvation. It is obvious that malnutrition affects the growth of infants. Furthermore, it has been shown that newborn babies experience what can be considered to be seriously starved conditions at birth (Burns et al., 2008, Adamkin and Committee on Fetus and Newborn, 2011), and that to survive these conditions, autophagy and milk play crucially important roles for the supply of nutrients as energy sources (Kuma et al., 2004). Our results indicate that milk, as a sugar source, is also necessary for the maturation of glycoproteins, which in turn contribute not only to the prevention of ER stress and cell death in many organs but also to the maintenance of growth-factor-mediated signaling for the normal post-natal growth of infants.

### Limitations of the Study

The study was conducted only in cultured mammalian cells. Therefore, for the substantiation of the beneficial functions of galactose during starvation *in vivo*, it will be necessary to perform animal experiments, examining both cellular and overall responses under similar conditions.

## Methods

All methods can be found in the accompanying Transparent Methods supplemental file.

## Supplemental Information

Document S1. Transparent Methods, Figures S1–S7, and Tables S1 and S2
