# Supplementary material for: A Trace Amount of Galactose, a Major Component of Milk Sugar, Allows Maturation of Glycoproteins during Sugar Starvation
Source: iScience. 2018 Nov 23;10:211–21. doi: 10.1016/j.isci.2018.11.035 (PMC6289946; doi:10.1016/j.isci.2018.11.035)
Supplement: Document S1. Transparent Methods, Figures S1–S7, and Tables S1 and S2 [file mmc1.pdf]

ISCI, Volume *10*

Supplemental Information

A Trace Amount of Galactose, a Major Component of Milk Sugar, Allows Maturation of Glycoproteins during Sugar Starvation

Norio Sasaoka, Hiromi Imamura, and Akira Kakizuka

Supplemental Information

A Trace Amount of Galactose, a Major Component of Milk Sugar, Allows Maturation of Glycoproteins During Sugar Starvation

Norio Sasaoka, Hiromi Imamura, Akira Kakizuka

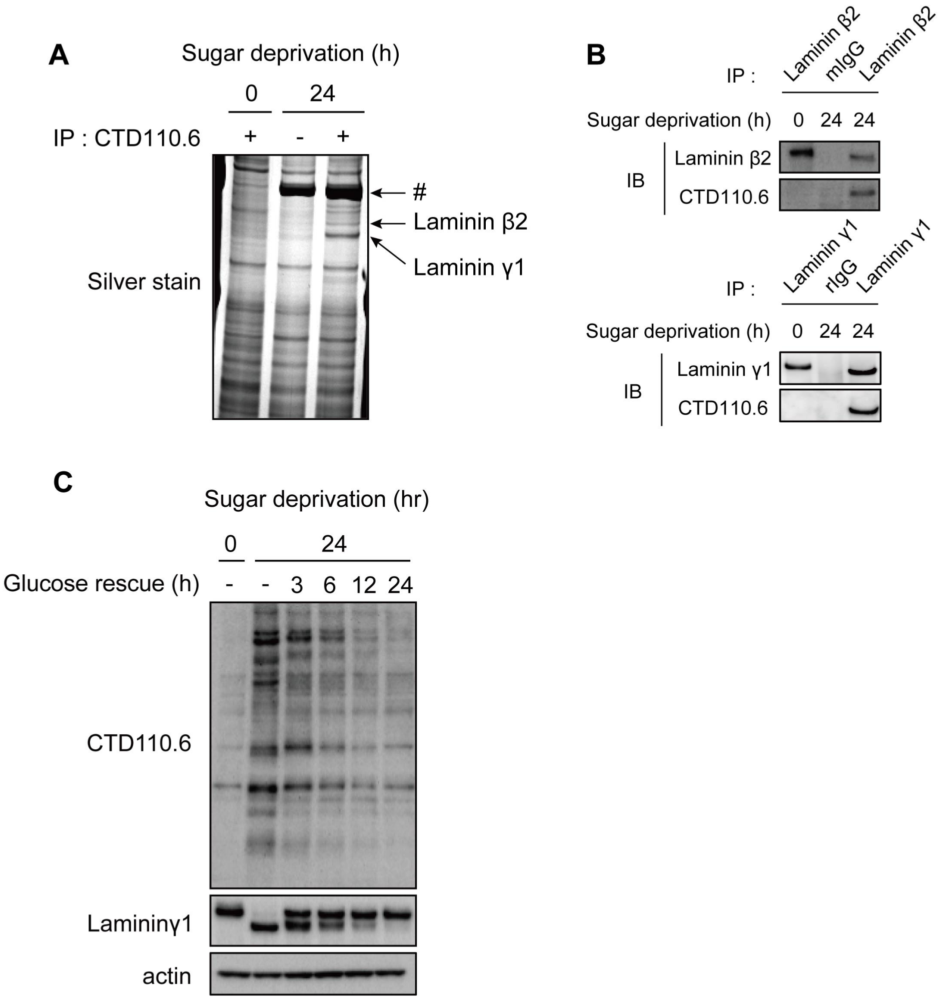

Figure S1. Identification of *N*-GlcNAc$_2$-Modified Proteins under Sugar-Free Conditions, Related to Figure 1.

(**A**) HEK293 cells were cultured in high-glucose medium (25 mM) or sugar-free medium (0 mM glucose) for 24 h. Proteins immunoprecipitated with CTD110.6 antibody were visualized on silver-stained gels. Laminin β2, Laminin γ1, and a non-specific signal (#) are shown. (**B**) HEK293 cells were treated as in **Figure 1A**, and anti-Laminin β2 or anti-Laminin γ1 immunoprecipitates were analyzed by western blotting with indicated antibodies. (**C**) HEK293 cells were grown with high-glucose medium, then deprived of glucose for 24 h. During this time, 3 mM glucose was added back to the medium for the indicated total times; thus, “24” indicates that glucose was added back to the medium immediately and remained in the medium over the full 24 h of subsequent growth, whereas “3” indicates that glucose was added back to the medium only 3 h before harvest. Western blots of cell lysates were probed with CTD110.6, anti-Laminin γ1, and anti-actin antibodies. Cells were cultured under serum-free conditions.

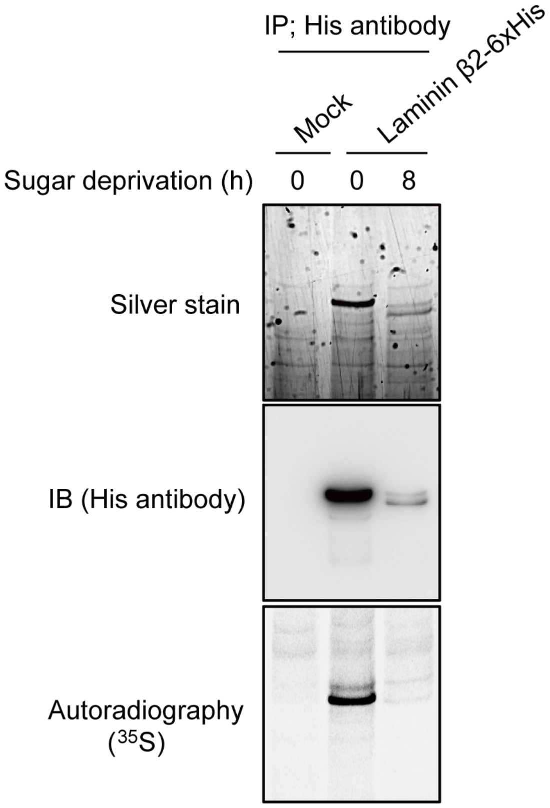

Figure S2. ***N*-GlcNAc$_2$-Modified Laminin β2 is a Newly Synthesized Product, Related to Figure 1.**

HEK293 cells were transfected with mock or C-terminal 6×His-tagged Laminin β2. At 24 h post-transfection, cells were cultured in serum-free medium containing 50 μCi of [^{35}S]methionine/cysteine (PerkinElmer) for 4 h. Following the pulse-labeling, cells were subjected to sugar deprivation for 8 h. Immunoprecipitates pulled down with an anti-His-tag antibody were analyzed by silver staining, western blotting, and autoradiography.

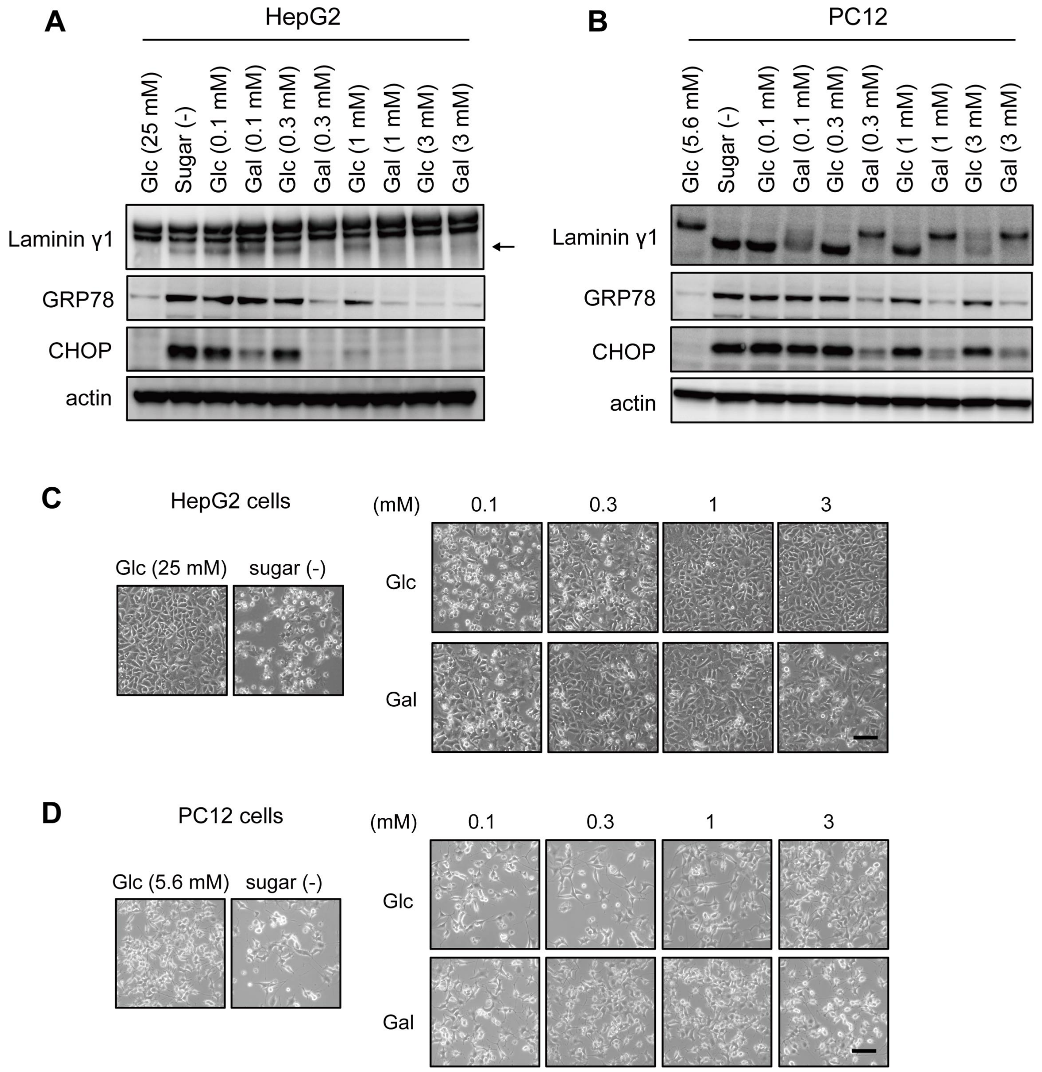

Figure S3. Galactose Preferentially Promotes Protein *N*-Glycosylation over Glucose in Low-Sugar Conditions in HepG2 and PC12 cells, Related to Figure 1. The effect of limiting amounts of galactose or glucose on *N*-glycosylation of Laminin γ1, and the ER stress marker CHOP, in HepG2 (**A**) and PC12 cells (**B**). In **A** and **B**, each immunoblot shows the Laminin γ1 cellular *N*-glycosylation level and CHOP as ER stress marker. The arrow indicates an immature lower molecular weight Laminin γ1 in HepG2 cells. Data are representative of three independent experiments. Photographs of HepG2 (**C**) and PC12 cells (**D**), cultured with the indicated concentrations of glucose or galactose for 8 h and 30 h, respectively. In PC12 cells, the culture medium was replaced with 50 ng/mL neurite growth factor in low-glucose DMEM with 1% FBS and 1% HS for 24 h prior to treatment. Scale bar, 50 μm. Cells were cultured under serum-free conditions.

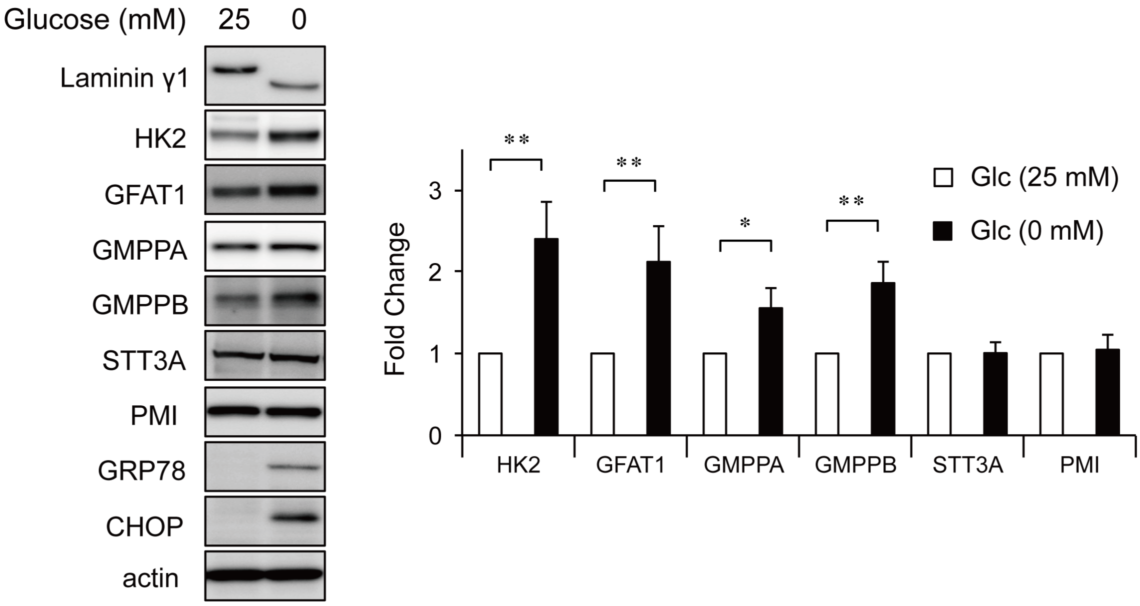

Figure S4. Expression Levels of *N*-Glycosylation-Related Proteins in HEK293 Cells, Comparing 25 mM Glucose to Sugar-Free Medium, Related to Figure 2. Expression levels of proteins related to sugar-free upregulated mRNA levels (see **Figures 2A-2C**) were analyzed by western blotting using the indicated antibodies (left panels). Cells were cultured under serum-free conditions. Quantitative analysis of the protein levels normalized to actin is shown (right panels). The data are the mean ± SEM (n = 3). * $P < 0.05$, ** $P < 0.01$ by Student's *t* test.

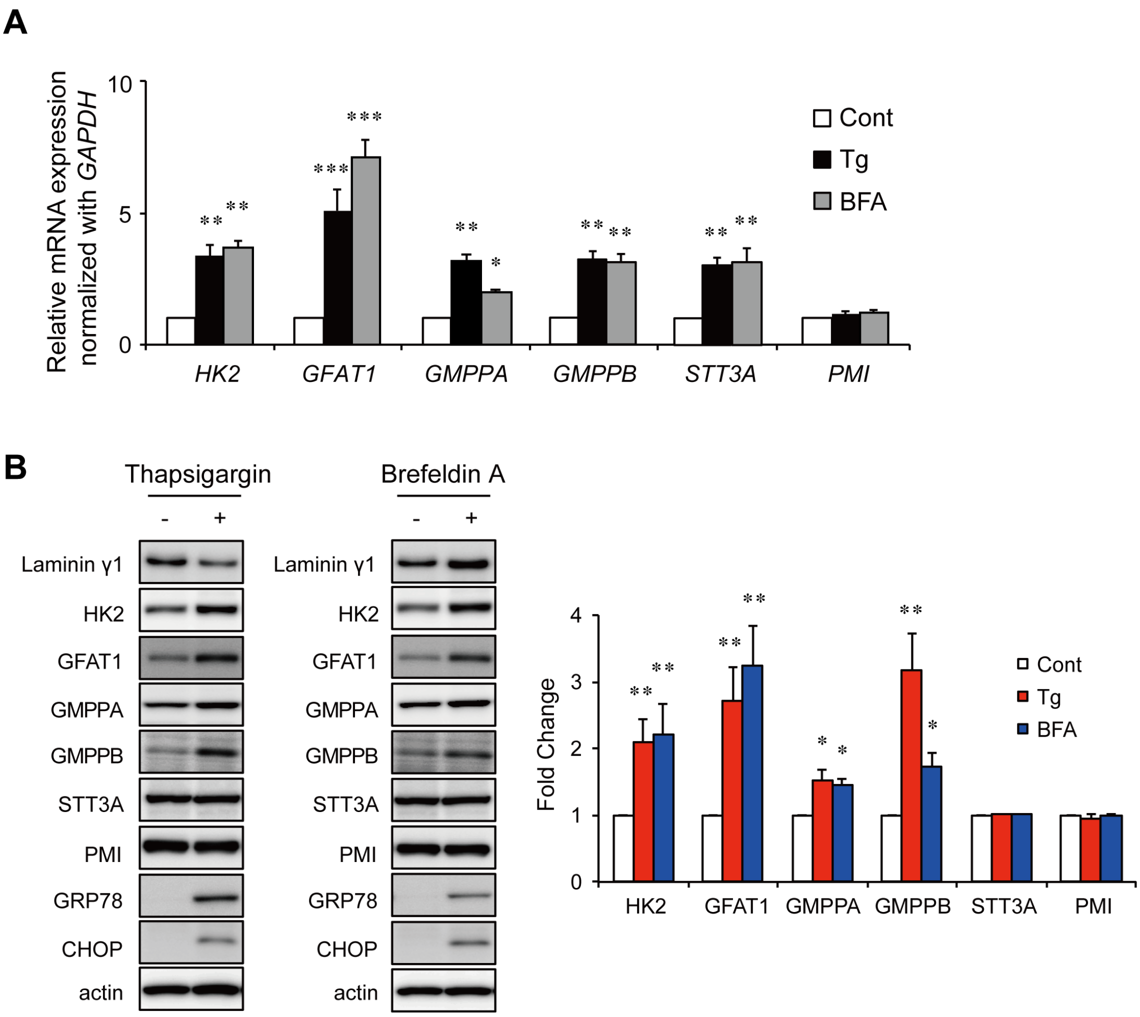

Figure S5. The ER Stress Inducers Thapsigargin and Brefeldin A also Upregulate *N*-Glycosylation-Related Genes, Related to Figure 2.

(**A**) RT-qPCR analysis of *N*-glycosylation-related genes in HEK293 cells after treatment with 0.1% DMSO (Cont, white color), 1 µM thapsigargin (Tg, black color) or 3 µg/mL brefeldin A (BFA, grey color) for 24 h. *PMI* was used as a negative control.
(**B**) Western blotting showing the levels of *N*-glycosylation-related proteins under the treatment conditions described in (**A**). Cells were cultured under serum-free conditions. Quantitative analysis of protein levels normalized to actin is shown (right panels). The data are the mean ± SEM (n = 3). * $P < 0.05$, ** $P < 0.01$ by Dunnett's test (vs. Cont.).

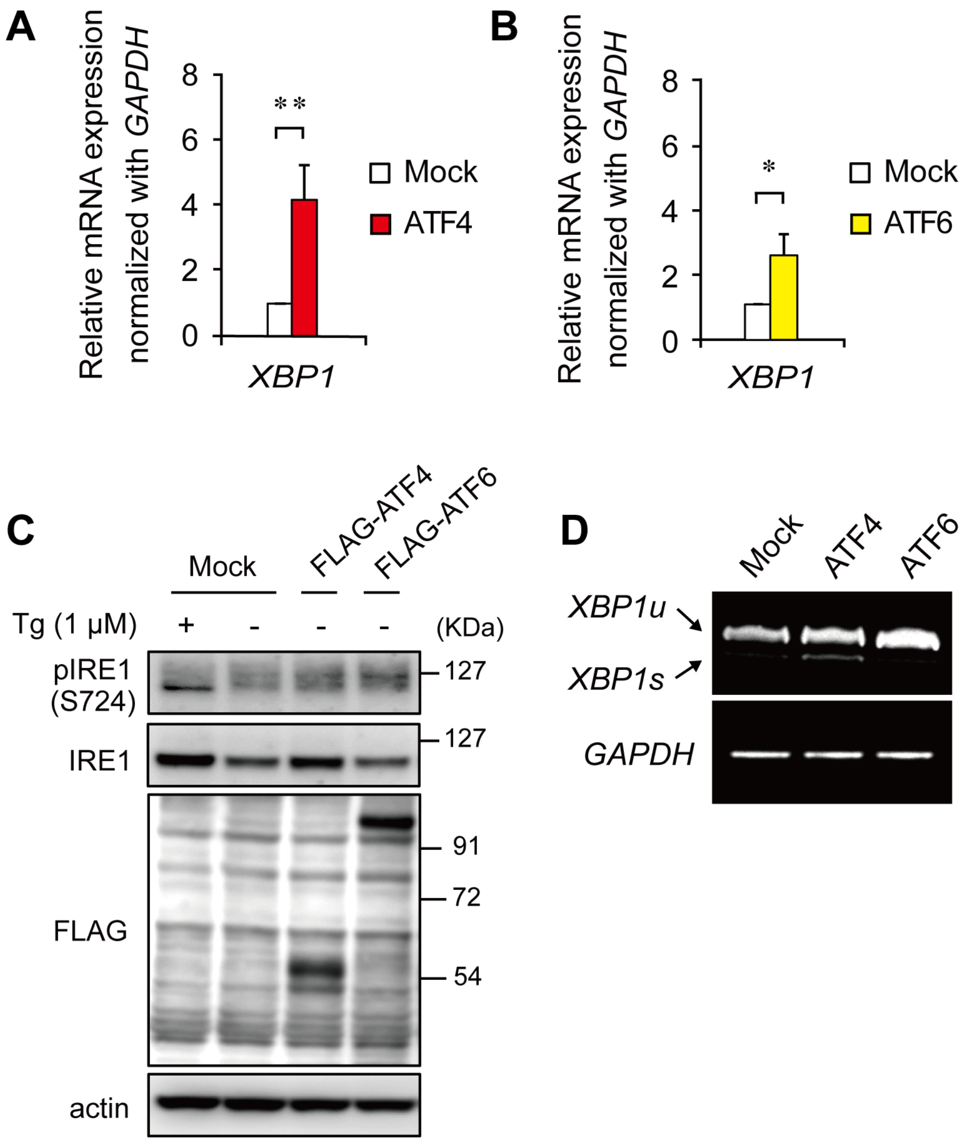

Figure S6. Expression of XBP1 was Induced by the Forced Expression of ATF4 or ATF6, Related to Figure 2.

(**A and B**) HEK293 cells were transfected with Mock and FLAG-ATF4 (**A**) or FLAG-ATF6 (**B**) for 24 h. The amount of *XBP1* was determined by RT-qPCR. The data are the mean ± SEM (n = 3). * $P < 0.05$, ** $P < 0.01$ by Student's *t* test. (**C**) HEK293 cells were transfected with empty (Mock), ATF4-, or an ATP6-expression vector and cultured for 48 h, followed by a 12h treatment with 0.1% DMSO or thapsigargin (Tg). Cell lysates were then subjected to western blotting analysis with the indicated antibodies. (**D**) Confirmation of overexpression efficacy of ATF4 or ATF6 in HEK293 cells by XBP1 splicing assay. Cells were cultured under serum-free conditions.

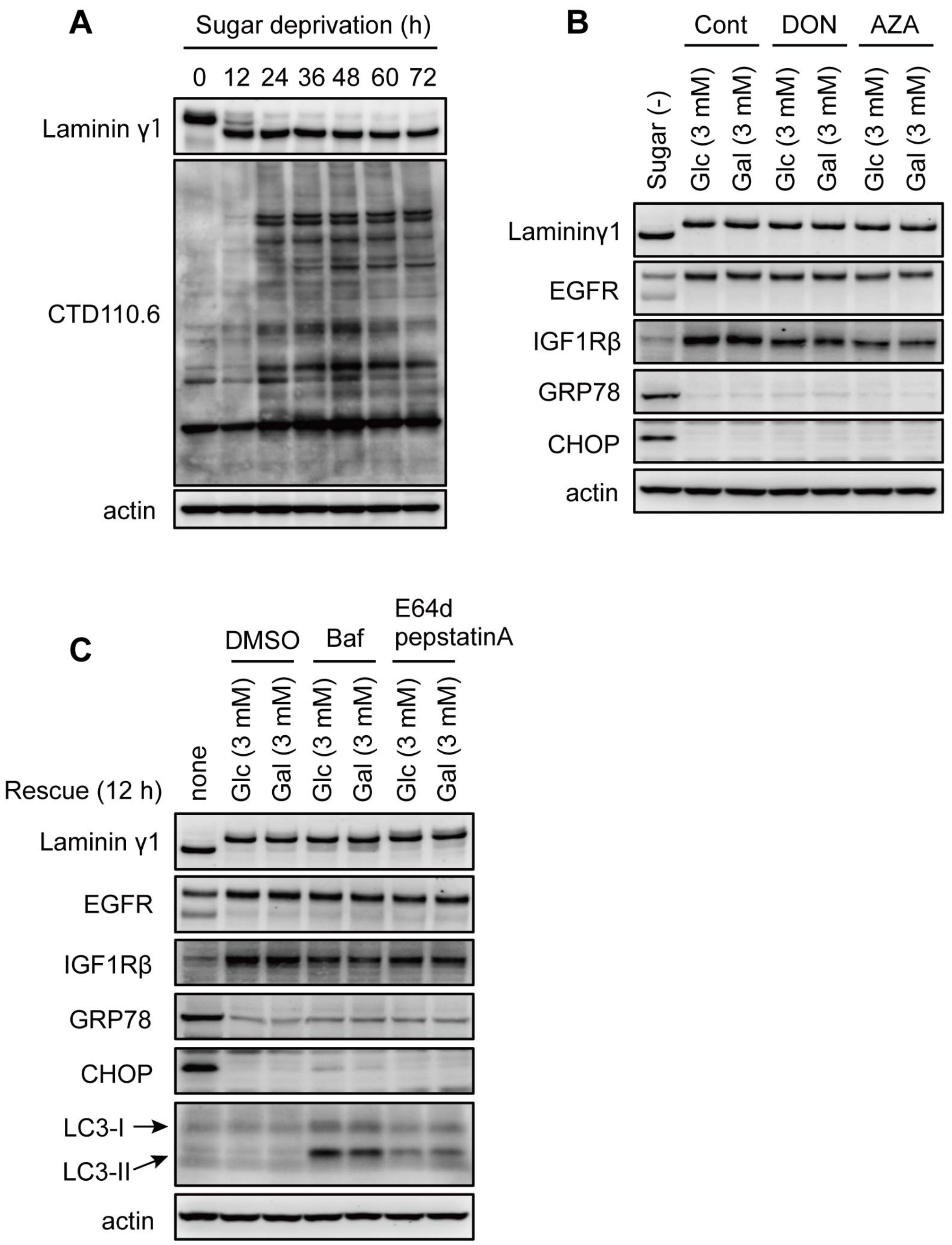

Figure S7. Maturation of Glycoproteins by Galactose is Independent of Autophagy, Related to Figure 3.

(**A**) Western blotting analysis of HEK293 cells cultured in the absence of sugar for the indicated times. Each immunoblot was probed with CTD110.6, anti-Laminin γ1, or anti-actin antibodies. (**B**) HEK293 cells cultured in medium containing 3 mM glucose or 3 mM galactose with or without HBP inhibitors (1 mM DON or 1 mM AZA) for 24 h were harvested, and then cell lysates were subjected to western blotting analysis with

the indicated antibodies. (**C**) HEK293 cells were cultured in glucose-free medium for 24 h, and then treated with medium containing 3 mM glucose or 3 mM galactose with or without autophagy inhibitors for 12 h. Inhibitors were bafilomycin A1 (1 μM, a lysosomal acidification inhibitor) and E64d/pepstatin A (10 μM each, both of which are lysosomal proteases inhibitors). Cell lysates were then analyzed by western blotting with the indicated antibodies. The conversion of LC3-I to LC3-II indicates autophagic activity. Cells were cultured under serum-free conditions.

Name of Primer	sequence (5' - 3')
EcoRI-mATF4-F	cggaattccATGACCGAGATGAGCTTCCTGAACAGC
mATF4-XbaI-R	gctctagaTTACGGAACTCTCTTCTTCCCCCTTGCC
ClaI-mATF6-F	ccatcgatcATGGTGGTGGTGGCAGCGGCGCCGAGC
mATF6-Asp718I-R	gctctagaTTAGACACTAATCAGCTGGGGGAAAAG
ClaI-mXBP1s-F	ccatcgatcATGGTGGTGGTGGCAGCGGCGCCGAGC
mXBP1s-XbaI-R	gctctagaTTAGACACTAATCAGCTGGGGGAAAAG

Table S1. List of Primers Used for Constructions, Related to Figure 2, S6, and Methods.

Gene	Forward sequence (5' - 3')	Reverse sequence (5' - 3')
Hexokinase 1 (HK1)	AGGTGAAGAAGAGGATGCGG	CGAAGGAGGGCAGCATCTTAAC
Hexokinase 2 (HK2)	GAGGATGAAGGTAGAAATGGAGCGA	GTAGCACACACGTAGGTGGG
Glucokinase (HK4)	CTACTACGAAGACCATCAGTGCGA	CATTCTGCATCTCCTCCATGTAGC
Glucose-6-phosphate isomerase (GPI)	GATGGACCAGCACTTCCGCA	TCTCACACCCAAAGCAGTTGAT
Phosphofructokinase, liver (PFKL)	TGACAAGAGGTTTGACGAGGCCA	TGGGCGAGGAGCTTGTAAAT
Phosphofructokinase, muscle (PFKM)	CCTGTGTAGTGAGCCTCTCTGGTAA	TCAGCTTCAGGGCTTCGTCAA
Phosphofructokinase, platelet (PFKP)	CGTGCAGATGACTCAGGATGT	TTGATGGCAAGTCGCTTGTAGG
Aldolase, fructose-bisphosphate A (ALDOA)	CGCAGGAGGAGTATGTCAAGC	ATAGGCGTGGTTAGAGACGAAGA
Aldolase, fructose-bisphosphate B (ALDOB)	GCTGCAAACAAGGAGGCAAC	CTGTGAAGAGCGACTGGGTG
Aldolase, fructose-bisphosphate C (ALDOC)	CTTACCTTCTCCTATGGGCGTG	CGCTTGATGAACTCCTCAGTGG
Triosephosphate isomerase 1 (TPI1)	AGCAGACAAAGGTCATCGCAGATAA	GGGTGTTGCAGTCTTGCCAGTA
Glyceraldehyde-3-phosphate dehydrogenase (GAPDH)	CCTGCACCACCAACTGCTTA	CAGTGATGGCATGGACTGTG
Phosphoglycerate kinase 1 (PGK1)	TCCGAGCTTCACTTTCCAAGCTA	TTCTGTGGCAGATTGACTCCTACC
Phosphoglycerate kinase 2 (PGK2)	CCGGATTCTTGATGAAGAAGGAAC	CTTGTCTGCCACTTTGGCTC
Phosphoglycerate mutase 1 (PGAM1)	GGAGGCGCTCCTATGATGTC	TCTTCTGTGAGGTCTGCATACCTG
Phosphoglycerate mutase 2 (PGAM2)	ACAGGCCTCAACAAGGCAGAAA	CCTTGCTAATGGAGTTGTAGTAGGG
Enolase 1 (ENO1)	AGAGGTTTACCACAACCTGAAGAAT	TCTCCAGGATGTTGGGAGCA
Enolase 2 (ENO2)	CAGTGGGAGCTGAGAGCTTT	GTGGCATCCTTGCCGTATTTG
Enolase 3 (ENO3)	GAGAACAATGAGGCCCTGGAG	AAGATCGTACTTCCCATTGCGATAG
Enolase 4 (ENO4)	CGAGATGCTTATGGAAATGCAGAA	AGTGATCTGCTGTTGACCTCTT
Lactate dehydrogenase A (LDHA)	AATTTGGTCCAGCGTAACGTGA	ATCTTCCAAGCCACGTAGGT
Lactate dehydrogenase B (LDHB)	CTAAGTGGATTACCCAAACACCGC	CTGCTGGGATGAATGCCAAG
Pyruvate kinase-Muscle (PKM2)	GGGTCGAGCAGGATGTTGATATG	ATGTTCTTTCCCTTCTCTCCCAG
Pyruvate kinase-Liver and RBC (PKLR)	GAAATCCTGGAGGTGAGCGAC	GCCCAATCATCATCTTCTGAGCC
Mannose phosphate isomerase (PMI)	TTCCGGCCAGTTGAGGAGATTGTAA	TCATGGCTCATGGTCTGCTT
Phosphomannomutase 1 (PMM1)	TTATGTGTTTGCCGAGAACGGG	CCAGGTGGTTCTGGATGGTC
Phosphomannomutase 2 (PMM2)	AGAAGAACGCATTGAGTTCTACGA	GCAAACTCTTTCCGTAGATCTGCT
Glutamine--fructose-6-phosphate transaminase 1 (GFAT1)	ATATTTGAGCAGCCAGAGTCTGTC	CCTTTATGTGATCCTTCAAACCACC
Glutamine--fructose-6-phosphate transaminase 2 (GFAT2)	ATACTATGAGAGGTCGGGTGAATTT	AACCATCACAGGAAGCTCAGTC
GDP-mannose pyrophosphorylase A (GMPPA)	AAACCCAGCACATTTATCAGTGAC	TGCCCATCCTGCTGATTACG
GDP-mannose pyrophosphorylase B (GMPPB)	GAGGAACCCTCCAAGTACGG	GCCTGCGTTGATCTTATTGGAC
Stt3a, subunit of the oligosaccharyltransferase complex (STT3A)	AGCTCCTCGTCTTCATGTTTCC	AGACTCCAATGCCAGAGAGAAT
Stt3b, subunit of the oligosaccharyltransferase complex (STT3B)	TGCAGGTGCTGTGTTCCTTAG	CCCAAGTCGTAGGTTGATGCTC
DNA damage inducible transcript 3 (CHOP)	CCAGCAGAGGTCACAAGCAC	ACTCTGTTTCCGTTTCCTGGTT
Heat shock protein family A (Hsp70) member 5 (GRP78)	CAACATGGATCTGTTCCGGTC	TTGGAATTCGAGTCGAGCCAC
X-box binding protein 1 (XBP1)	TTACGAGAGAAAACTCATGGCC	GGGTCCAAGTTGTCCAGAATGC
eukaryotic translation initiation factor 2 alpha kinase 3 (PERK)	CATCCTCATCCTCACAGGCAAAG	TCATTCCAGCTACTGTCATTGGC
endoplasmic reticulum to nucleus signaling 1 (IRE1)	GGTGGCCTTCATCATCACCTAT	CTGCAGGAGCTGGATCTTCT
activating transcription factor 6 (ATF6)	CACCCACTAAAGGCCAGACG	CGTGATTAGGGAGCTGTGTGAC

Table S2. List of Primers Used for RT-qPCR, Related to Figure 2, S5, S6, and Methods.

TRANSPARENT METHODS

Cell Cultures and Plasmid Transfection

HEK293 and HepG2 cells were cultured in high-glucose Dulbecco's modified Eagle's medium (DMEM, Nacalai Tesque), and supplemented with 10% fetal bovine serum (FBS) at 37ºC in 5% CO_2. PC12 cells were maintained in low-glucose DMEM (5.6 mM glucose) with 10% FBS and 5% horse serum. Cells were initially seeded in each medium, washed once with PBS and cultured in DMEM without glucose (Nacalai Tesque, referred as "sugar-free") supplemented with various concentrations of monosaccharides (glucose, mannose, and galactose) without FBS. When needed, HEK293 cells were transfected with expression plasmids using Lipofectamine LTX with Plus reagent (Invitrogen), according to the manufacturer's protocol.

Construction of Expression Vectors

Mouse full-length ATF4, ATF6, and spliced XBP1 (XBP1s) were amplified from cDNA from B16F10 cells using the indicated primers (see Table S1), and subcloned into EcoRI-XbaI sites, ClaI-Asp718I sites, and ClaI-XbaI sites of the pFLAG-CMV2 vector, respectively. C-terminal 6×His-tagged rat Laminin β2 was obtained by subcloning the cDNA insert into the pcDNA3-6×His vector.

Reagents

D-glucose, D-mannose, D-galactose, L-glutamine, sodium pyruvate, and 2-deoxy-D-glucose (2-DG) were purchased from Nacalai Tesque. Thapsigargin (Tg) was obtained from WAKO. Rotenone, oligomycin, bafilomycin A1, E64d, pepstatin A, and UDP-Galactose (UDP-Gal) were purchased from Sigma-Aldrich. Brefeldin A (BFA) and galactose-1-phosphate (Gal-1P) were obtained from Millipore. Azaserine (AZA) and 6-diazo-5-oxo-1-norleucine (DON) were purchased from Santa Cruz Biotechnology.

Western Blotting

Cells were lysed in RIPA buffer (150 mM NaCl, 1% Nonidet P-40, 1% sodium deoxycholate, 0.1% SDS, 25 mM Tris-HCl, pH 7.6) containing a protease inhibitor cocktail (Roche), sonicated, and centrifuged at 15,000 ×*g* for 10 min. The protein content of supernatants was quantified using a BCA assay kit (Nacalai Tesque). Proteins

were separated on pre-cast 5–20% gradient polyacrylamide gels (WAKO) and transferred to polyvinylidene difluoride membranes (Millipore). The membranes were then incubated overnight with primary antibodies, described below. ECL anti-rabbit or anti-mouse IgG HRP-linked whole antibody (GE Healthcare, 1:2,500) was used as the secondary antibody, and signals were detected using ECL Western blotting detection reagent (GE Healthcare) and ImageQuant LAS4000 (GE Healthcare). Protein bands were quantified by densitometry analysis using ImageJ software. Actin was used as a loading control.

Antibodies

Antibodies against Laminin γ1 (sc-5584) and CHOP (sc-793) were purchased from Santa Cruz Biotechnology. Laminin β2 antibody (C4) was kindly provided by Dr. Kikkawa (Tokyo University of Pharmacy and Life Sciences, Japan). Antibodies against HK2 (C64G5), GRP78 (C50B12), MEK1/2 (47E6), Histone H3 (D1H2), ERK (9102), phospho-ERK (9101), IRE1α (14C10), EGFR (D38B1), and IGF1Rβ (D23H3) were purchased from Cell Signaling. Antibodies against GFAT1 (EPR4854), PMI (EPR10234), phospho-IRE1α (EPR5253), and PFKP (1D6) were obtained from Abcam. The antibody against actin (clone C4) was from Chemicon. Anti-*O*-GlcNAc (CTD110.6) antibody was obtained from Covance. Anti-His (OGHis) and anti-LC3 (PM036) antibodies were purchased from MBL. Anti-GMPPA (15517-1-AP), anti-GMPPB (15094-1-AP), and anti-STT3A (12034-1-AP) antibodies were obtained from Proteintech. Anti-FLAG (M2) antibody was purchased from Sigma-Aldrich.

Immunoprecipitation

HEK293 cells were lysed in lysis buffer (150 mM NaCl, 1% Nonidet P-40, 5% glycerol, 1 mM EDTA, 25 mM Tris-HCl, pH 7.4) containing protease inhibitor cocktail and incubated for 2 h at 4°C with CTD110.6 antibody (1:1,000), Laminin γ1 antibody (1:1,000), or Laminin β2 antibody (1:1,000). The immunocomplexes were bound to Protein A/G PLUS-agarose (Santa Cruz) overnight at 4°C and washed five times with TBS. For LC-MS/MS analysis, the immunocomplexes were eluted with 0.1 M glycine-HCl (pH 3.0) and immediately neutralized with 2 M Trizma base, and for other experiments the immunocomplexes were eluted with 1× SDS sample buffer and heat-denatured at 90°C for 5 min.

LC-MS/MS Analysis and Identification of *N*-GlcNAc$_2$ Modified Proteins

To identify the CTD110.6-interacting bands, gels were silver-stained using Sil-Best Stain One (Nacalai Tesque) and immunoblotted with CTD110.6 antibody. Each identified band was digested in the gel by modified trypsin (Promega). The extracted samples were analyzed by LC-MS/MS using an LTQ mass spectrometer (Thermo Fisher Scientific) combined with Paradigm MS4 HPLC (Michrom BioResources). Both MS and MS/MS spectra were searched against the Mascot database (Matrix Science) to identify the proteins.

RT-qPCR

Total RNA was isolated from cells using the SV total RNA isolation kit (Promega). For cDNA synthesis, total RNA (1 μg) was reverse transcribed using ReverTra Ace qPCR RT Master Mix with gDNA Remover (TOYOBO), according to the manufacturer's protocol. qPCR was performed on a LightCycler PCR System (Roche Diagnostics) and carried out at 98ºC for an initial 2 min followed by 45 cycles of denaturation at 98ºC for 10 sec, annealing at 60ºC for 10 sec, and extension at 68ºC for 30 sec using KOD SYBR qPCR Mix (TOYOBO). Primer sets are listed in Table S2. Primers were designed and selected with the use of LightCycler Probe Design software 2.0 (Roche Applied Bioscience). The *GAPDH* was used as an internal control for normalization of target gene expression.

XBP1 splicing assay

To assess the variant *XBP1* by PCR, sequence of the primers were used as same as qPCR, corresponding to nucleotides 428-449 and 689-710 of the human *XBP1* cDNA generating two PCR products of 283 (unspliced *XBP1*, *XBP1u*) and 257 (spliced *XBP1*, *XBP1s*) base pairs. Cycling condition was carried out at 95ºC for an initial 30 sec followed by 28 cycles of denaturation at 95ºC for 10 sec, annealing at 60ºC for 10 sec and extension at 72ºC for 5 sec using PrimeSTAR Max DNA polymerase (TaKaRa). The PCR products were separated on a polyaclylamide gel. The *GAPDH* was used as an internal control.

RNA Interference

siRNAs for *PERK* (s18102), *IRE1* (s200430), *ATF6* (s22688), and *XBP1* (s14913) were obtained from Ambion (LifeTechnologies) using the Silencer Select siRNA series. Target siRNA or control siRNA (Silencer Select Negative Control No.1 siRNA; Ambion) was transfected into HEK293 cells using Oligofectamine (Invitrogen) in each well of 6-well plates following the manufacturer's instructions. The cells were cultured for 72 h prior to use in the knockdown experiments. .

ECAR and OCR Measurements

ECAR and OCR were measured using the Seahorse XF96 Flux Analyzer in accordance with the manufacturer's instructions (Seahorse Bioscience, USA). The XF96 microplate was coated with type I collagen (Nitta Gelatin) and left for at least 1 h at room temperature. Briefly, cells were seeded in a microplate in the corresponding cell growth medium and then incubated at 37ºC in 5% CO_2 for 24 h. Before changing the assay medium, cells were exposed to sugar-free conditions for 3 h. Assays were initiated by replacing the medium with 175 μL assay medium supplemented with 4 mM glutamine and without sugars. The microplates were incubated at 37ºC for 2 h to equilibrate the temperature and pH of the media before measurement. After incubation, the plate was loaded on the analyzer. The concentrations of compounds sequentially added to the medium (175 μL) of the cell plate during the course of the experiment were 24 mM sugar, 27 μM rotenone, and 1 M 2-DG in volumes of 25 μL each, resulting in final concentrations of 3 mM sugar, 3 μM rotenone, and 100 mM 2-DG, respectively. The final injection was 2-DG, a glucose analog, which inhibits glycolysis through competitive binding to glucose hexokinase, the first enzyme in the glycolytic pathway. All values of ECAR and OCR were normalized to cell number. Data shown are the representative mean ± SEM from three independent experiments.

Carbon-14 Labeling Study

D-[1-^{14}C]galactose (PerkinElmer) was used for labeling sugar chains on 6×His-tagged Laminin β2. Cells transfected with mock or a 6×His-tagged Laminin β2 expression construct were plated in 6-well plates, and maintained for 24 h. When cells were 100% confluent, the medium was changed to sugar-free medium or 0.3 mM [1-^{14}C]galactose medium for 24 h. After cells were harvested and lysed, 6×His-tagged Laminin β2 was

pulled down using a His-tagged Protein PURIFICATION KIT (MBL), followed by SDS-PAGE and autoradiography. Detection of the labeled Laminin β2 by autoradiography was performed using an FLA7000 image analyzer (Fujifilm).

Metabolomic Analysis

HEK293 cells were seeded in three 10 cm dishes and incubated in growth media until they reached 90% confluence, then treated with medium that was sugar-free, or supplemented with 3 mM glucose or 3 mM galactose, for 6 h. The respective media were removed from the plates, and cells were washed twice with 5% mannitol solution (10 mL first and then 2 mL), treated with 800 μL of methanol (WAKO), and let stand for 30 sec. The cell extract was then treated with 550 μL of Milli-Q water containing internal standards (Human Metabolome Technologies (HMT)) and let stand for another 30 sec. The extract was obtained and centrifuged at 2,300 ×*g* and 4°C for 5 min, and then 800 μL of the upper aqueous layer was centrifugally filtered through a Millipore 5-kDa cutoff filter (UltrafreeMC-PLHCC, HMT) to remove macromolecules (9,100 ×*g*, 4°C, 120 min). The filtrate was centrifugally concentrated and resuspended in 50 μL of Milli-Q water for metabolome analysis at HMT and analyzed by a CE-TOFMS system (Agilent Technologies). Signal peaks were annotated according to the HMT metabolite database based on their m/z values with the migration times. Detected metabolites were plotted on metabolic pathway maps using VANTED software.

Spectrophotometric PFK1 Activity Assay

PFK1 activity was measured with slight modifications to the described procedure (Deng et al., 2008). Cells were washed with 1 × PBS, pH 7.4, and incubated in lysis buffer (50 mM Tris–HCl, pH 7.5, 1 mM EDTA, 150 mM NaCl, 1% Nonidet P-40, 0.5 mM DTT, protease inhibitor cocktail) for 10 min on ice. Briefly, the assay was performed by adding 5 μg cell lysate to 100 μL of reaction buffer containing 50 mM Tris-HCl pH 7.5, 100 mM KCl, 5 mM $MgCl_2$, 1 mM ATP, 0.2 mM NADH (Nacalai Tesque), 5 mM Na_2HPO_4, 0.1 mM AMP, 1 mM NH_4Cl, 5 mM fructose-6-phosphate, 5 units/mL of triose phosphate isomerase (Sigma-Aldrich), 1 unit/mL of aldolase (Sigma-Aldrich), and 1 unit/mL of α-glycerophosphate dehydrogenase (Sigma-Aldrich). The mixture was incubated at room temperature, and absorbance at 340 nm, which reflects the NADH

concentration, was determined each minute for up to 20 min using a DU640 spectrophotometer (Beckman).

Statistical Analysis

Each experiment was conducted at least three times with consistent results. The gel or blot representative of each experiment is presented. The statistical significance was analyzed using Student's *t* test or Dunnett's test.

SUPPLEMENTAL REFERENCE

Deng, H., Yu, F., Chen, J., Zhao, Y., Xiang, J., and Lin, A. (2008). Phosphorylation of Bad at Thr-201 by JNK1 promotes glycolysis through activation of phosphofructokinase-1. J. Biol. Chem. *283*, 20754-20760.
